# Integrated multi-month dispensing of antihypertensive and antiretroviral therapy to sustain hypertension and HIV control

**DOI:** 10.1038/s41371-022-00655-3

**Published:** 2022-03-04

**Authors:** Isaac Derickk Kimera, Christabellah Namugenyi, Jeremy I. Schwartz, Douglas Joseph Musimbaggo, Rebecca Ssenyonjo, Praise Atukunda, Gerald Mutungi, Frank Mugabe, Fortunate Ambangira, Mary Mbuliro, Rodgers Katwesigye, Dinesh Neupane, Isaac Ssinabulya, Fred Collins Semitala, Christian Delles, Martin Muddu

**Affiliations:** 1 Makerere University Joint AIDS Program, Kampala, Uganda; 2 Section of General Internal Medicine, Yale School of Medicine, 333 Cedar Street, New Haven, CT 06511 USA; 3 Uganda Initiative for Integrated Management of Non-Communicable Diseases (UINCD), Kampala, Uganda; 4 Department of non-communicable diseases, Uganda Ministry of Health, Kampala, Uganda; 5 Johns Hopkins Bloomberg School of Public Health, Baltimore, MD USA; 6 Lancet Commission on Hypertension Group, Atlanta, GA USA; 7 Makerere University College of Health Sciences, Kampala, Uganda; 8 Uganda Heart Institute, Kampala, Uganda; 9 Institute of Cardiovascular and Medical Sciences, University of Glasgow, Glasgow, UK

**Keywords:** Hypertension, Cardiovascular diseases

## Abstract

Multi-month dispensing (MMD) is a patient-centered approach in which stable patients receive medicine refills of three months or more. In this pre-post longitudinal study, we determined hypertension and HIV treatment outcomes in a cohort of hypertensive PLHIV at baseline and 12 months of receiving integrated MMD. At each clinical encounter, one healthcare provider attended to both hypertension and HIV needs of each patient in an HIV clinic. Among the 1,082 patients who received MMD, the mean age was 51 (SD = 9) years and 677 (63%) were female. At the start of MMD, 1,071(98.9%) patients had achieved HIV viral suppression, and 767 (73.5%) had achieved hypertension control. Mean blood pressure reduced from 135/87 (SD = 15.6/15.2) mmHg at the start of MMD to 132/86 (SD = 15.2/10.5) mmHg at 12 months (*p* < 0.0001). Hypertension control improved from 73.5% to 78.5% (*p* = 0.01) without a significant difference in the proportion of patients with HIV viral suppression at baseline and at 12 months, 98.9% vs 99.0% (*p* = 0.65). Patients who received MMD with elevated systolic blood pressure at baseline were less likely to have controlled blood pressure at 12 months (OR-0.9, 95% CI, 0.90,0.92). Overall, 1,043 (96.4%) patients were retained at 12 months. Integrated MMD for stable hypertensive PLHIV improved hypertension control and sustained optimal HIV viral suppression and retention of patients in care. Therefore, it is feasible to provide integrated MMD for both hypertension and HIV treatment and achieve dual control in the setting of sub-Saharan Africa.

## Introduction

Sub-Saharan Africa (SSA) is faced with a dual burden of cardiovascular and infectious diseases [1]. Uganda has approximately 1.5 million persons living with HIV (PLHIV) of whom over 90% are on antiretroviral therapy (ART) [2]. Even though HIV mortality has reduced by 50% in the past 20 years due to ART, PLHIV increasingly suffer from cardiovascular diseases––of which uncontrolled hypertension is a major contributor [1]. In Uganda, about a quarter of adult PLHIV have hypertension [3, 4]. Despite the high burden of hypertension, SSA has low levels of hypertension awareness, treatment, and control [5]. Hypertension control in SSA is less than 20% [6, 7]. The key barriers to hypertension control in SSA include a shortage of healthcare professionals [8], high rates of loss to follow-up [9], and poor access to hypertension medicines [10]. This calls for innovative measures to address the dual burden of hypertension and HIV/AIDS in SSA. One potential innovation is to integrate the management of hypertension into HIV clinics while leveraging the HIV program gains [11]. For successful integration of hypertension and HIV care, it is necessary to adopt HIV best practices like improved access to medicines, longer medicines refills for stable patients, and task shifting.

In Uganda’s HIV program, multi-month dispensing (MMD) was adopted from the World Health Organization, Uganda National Guidelines for HIV [12], and The President’s Emergency Plan For AIDS Relief (PEPFAR) guidance [13]. Multi-month dispensing is implemented as a patient-centered approach in which stable patients (with HIV viral suppression and good drug adherence) receive ART refills of three to six months as opposed to monthly. Multi-month dispensing holds great potential for expansion to other chronic health conditions.

MMD has been shown to reduce the number of clinic visits, patients’ travel and time costs, decongests HIV clinics, reduces the total time spent in clinics by 50%, and improve retention. MMD also reduces the number of prescriptions without a change in the number of patients served, thus reducing human resource fatigue [14–18].

However, the feasibility and effectiveness of integrating MMD of hypertension medications and ART among hypertensive PLHIV have not been studied in SSA, where monthly dispensing of hypertension medicine is the usual practice [19, 20]. In this study, we determined treatment outcomes among hypertensive PLHIV who received integrated MMD of hypertension and HIV medicines at Uganda’s largest HIV clinic at baseline and after 12 months of MMD.

## Methods

### Study design

We conducted a pre-post, prospective longitudinal study among a cohort of hypertensive PLHIV enrolled into integrated facility-based multi-month dispensing of hypertension and ART medicines by doctors, clinical officers, and nurses at Mulago Immune Suppression Syndrome (ISS) clinic from August 2019 to March 2021.

### Study setting

Mulago ISS is a large HIV clinic located within Mulago National Referral Hospital complex in Kampala, Uganda. The HIV clinic is owned and operated by a non-governmental organization, Makerere University Joint AIDS Program (MJAP). The clinic provides comprehensive HIV services to over 16,500 PLHIV. Following a two-year project funding from Resolve to Save Lives [21], a US-based non-governmental organization that supports countries to utilize evidence-based strategies to improve hypertension control, Mulago ISS clinic began to provide integrated hypertension and HIV services in August 2019.

### Inclusion criteria

We followed up patients in a cohort that had received integrated MMD of hypertension and ART medicines between August 2019 and March 2020, to assess the hypertension and HIV treatment outcomes. We chose this period because it enabled us to follow-up the last entrant for 12 months. Participants were receiving both ART and hypertension medication.

### Study participants

These were hypertensive PLHIV receiving integrated MMD of hypertension and HIV treatment at the clinic. The patients needed to have a suppressed HIV viral load and good adherence (HIV stable) with controlled hypertension before they received MMD. However, HIV stable patients with uncontrolled hypertension would be given MMD at the discretion of the health provider when they requested.

We defined controlled hypertension as both systolic blood pressure <140 mmHg and diastolic blood pressure <90 mmHg, while controlled HIV as serum viral load of <1000 copies/ml.

### Implementation package

To integrate hypertension care into the HIV clinic, we implemented a package of five major components of, (1) Healthy lifestyle counseling, (2) Use of a step-wise treatment protocol, (3) Access to both ART and hypertension medicines to patients at no cost, (4) Team-based care including task shifting and (5) Systems for monitoring integrated hypertension and HIV care (Table 1).

**Table 1:** Key elements of the implementation package for integrated hypertension and HIV care.

Package component	Description
Healthy lifestyle counseling	Counseling on healthy lifestyle: smoking cessation, low salt intake, regular physical exercise, healthy diet, and ART adherence by HIV Peer counselor, nurses and clinicians.
A step-wise hypertension treatment protocol	Developed a single page, step-wise treatment protocol to guide integrated hypertension and HIV treatment.
Trainings	Trained all the healthcare providers at the clinic on the adapted and adopted WHO HEARTS-based protocol for hypertension management.
	The trainings were delivered as part of the clinic’s schedule of continuous medical education sessions.
Access to hypertension medicine and blood pressure machines	Procured and provided both and hypertension medicines to patients at no cost, and ART was supplied through the national supply chain systems.
	Procured two additional Omron-M6 validated automated blood pressure machines to supplement the existing ones in the HIV clinic.
Team-based care and task shifting	Peer counselors/expert clients screened for hypertension and provided healthy lifestyle and drug adherence counseling.
	Nurses supplemented clinical officers and doctors to prescribe both hypertension medicine and ART for patients with no medical complaints.
Systems for monitoring	Developed a hypertension register and an electronic database for integrated hypertension and HIV information.

### Hypertension medicine regimens

**Fig. 1:**
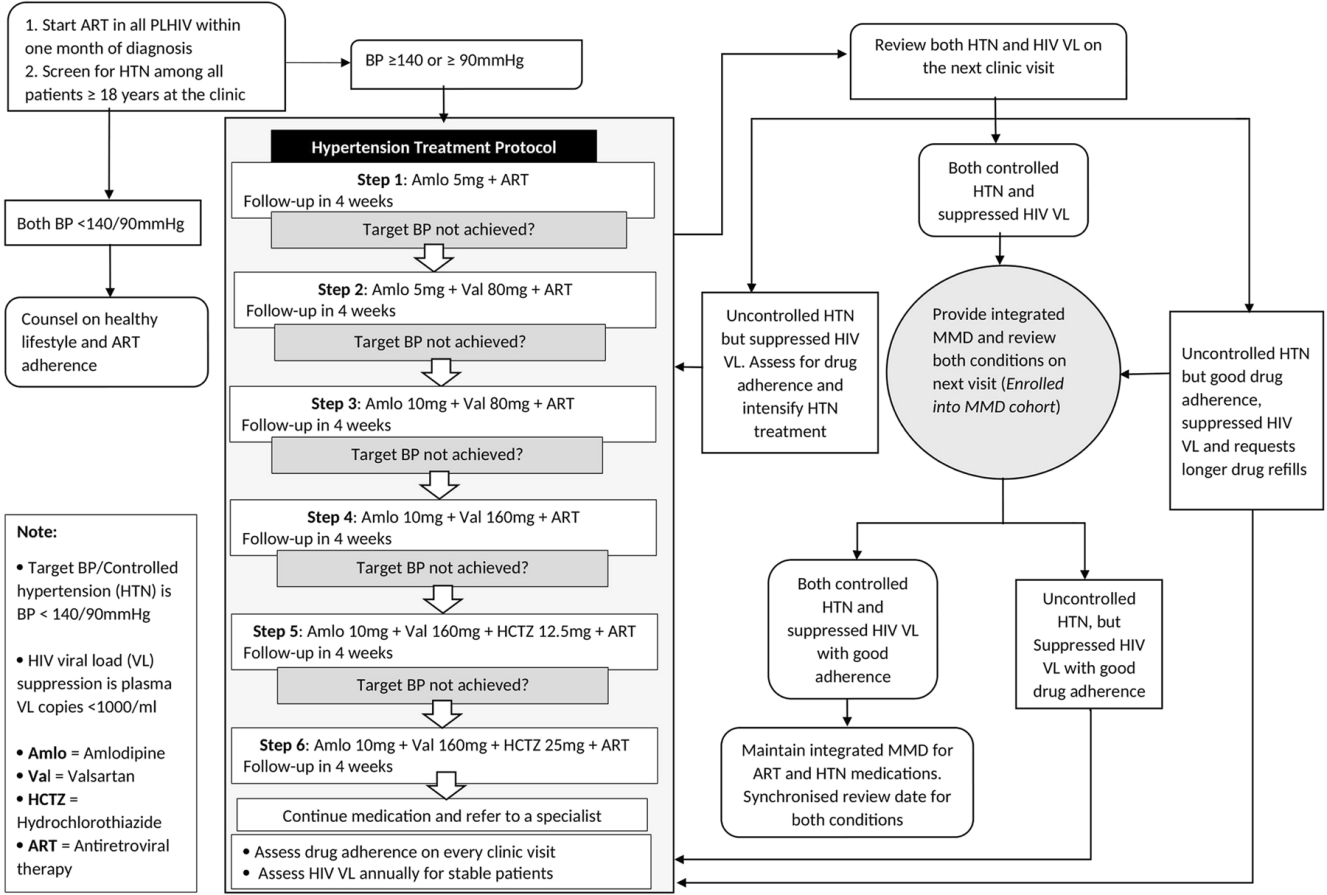
Algorithm for integrated management of hypertension and HIV, and patient entry into integrated multi-month dispensing. The first and second panels describe screening, diagnosis and treatment of hypertension in the HIV clinic, while the third panel describes the criteria for receiving integrated multi-month dispensing. HTN hypertension, Amlo amlodipine, Val valsartan, HCTZ hydrochlorothiazide, VL viral load, ART anti-retroviral therapy.

We procured hypertension medicines (amlodipine, valsartan and hydrochlorothiazide) from a private-not-for-profit medicine access program that has a memorandum of understanding with the Uganda Ministry of Health to provide low cost, quality medicines for non-communicable diseases including hypertension. We provided hypertension medicines to patients at no cost. Our choice of medicines was based on local efficacy and availability. Local evidence in Uganda suggests that combination therapy of long-acting dihydropyridine calcium-channel blocker like amlodipine and an angiotensin converting enzyme inhibitor are efficacious for hypertension treatment in black Africans [22]. We arranged the hypertension medicines into six steps of progressive treatment intensification on the hypertension treatment protocol (Fig. 1).

### Participant enrollment into integrated MMD for hypertension and ART medications

All PLHIV are initiated on ART within one month of HIV diagnosis according to the test and treat policy [12]. All adult PLHIV ≥18 years at the clinic were screened for hypertension, and once diagnosed, they were initiated on hypertension treatment per the protocol. We measured blood pressure after five minutes of rest, in a sitting position for all patients. For blood pressures ≥140/90 mmHg, we measured blood pressure three times with a one-minute interval and took the lowest reading of the three as the final record. We used Omron-M6 (HEM-7321-E) validated automated blood pressure machines for the baseline and follow-up visits [23]. For patients who had persistently elevated blood pressures ≥140/90 mmHg, we reviewed their previous blood pressure values in the electronic medical records at least a month earlier before diagnosing hypertension and recommending treatment. Hypertensive PLHIV received integrated monthly medicine refills for both hypertension and ART, with hypertension treatment intensification until their blood pressure was controlled. HIV viral load was monitored after six months for newly diagnosed PLHIV and annually for stable patients. Patients who achieved both HIV and hypertension control received MMD of three months. In addition, patients who had achieved HIV control but with uncontrolled hypertension and good treatment adherence were given MMD if requested. Main reason to request for MMD among such patients was high transport costs of monthly appointments and a need to travel. Patients enrolled on integrated MMD who returned with blood pressure ≥140/90 mmHg (with good drug adherence), were maintained on the same service delivery model, based on the decision of the healthcare provider, but had their hypertension treatment intensified as per the treatment protocol. However, patients who returned with unsuppressed HIV viral load received intensified adherence counseling and integrated monthly drug refills (Fig. 1). At each clinical encounter, the same clinician addressed both hypertension and HIV needs of each patient: provided treatment adherence support, prescribed both hypertension medicines and ART, and gave the same date to review both HIV and hypertension.

### Data collection

Data were collected during routine clinic visits of enrolled participants on body mass index, blood pressure values, hypertension medicines regimens, and hypertension adverse drug events. For HIV, we collected data on ART regimen, adverse drug events, and HIV viral suppression. These data elements were recorded in the patients’ files and in the electronic medical records (EMR) by the attending health worker. A records officer transferred the data from patient files to a Microsoft Access database. All the data sources (patient files, EMR, and Microsoft access database) were reviewed daily for completeness.

### Statistical analysis

For baseline characteristics of participants, we used the Pearson chi-square and Analysis of Variance (ANOVA) tests to determine significant association between gender and the following parameters: age, baseline mean systolic and diastolic blood pressure, baseline hypertension control, HIV control, ART regimen, duration on ART, body mass index and comorbidities like diabetes mellitus (using fasting blood glucose) and chronic kidney disease.

ANOVA was used to determine if there was a significant difference between systolic and diastolic blood pressure at baseline and at the end of each participant study period (12 months). Pearson chi-squared and Fisher’s exact tests were used to determine if there was a significant difference between hypertension and HIV control at baseline and at 12 months for each participant.

We obtained adjusted Odds Ratios using a multivariable random effects logistic model to assess the predictors of hypertension control over 12 months among patients who received MMD.

We plotted the quarterly longitudinal trend of HIV and hypertension control during the 12 months of follow-up. We conducted all analyses in STATA version 14.

## Results

Between August 2019 and March 31st 2020, 1082 (95%) of all patients who received integrated hypertension and HIV care at the clinic had been initiated on MMD. We followed this cohort for 12 months, until March 31st 2021. All the patients who had not been initiated on MMD were receiving monthly dispensing for uncontrolled hypertension, having been recently enrolled on integrated hypertension and HIV care.

At the start of MMD, females had higher rates of hypertension control, and lower average systolic blood pressures than their male counterparts (77.1% vs 67.6%) and (133/87 vs 138/87 mmHg) respectively. Majority of the women 682 (63%) had a body mass index (BMI) > 25 kg/m², compared to 400 (40%) of the men. More than 80% of the patients had been on ART for more than five years. The prevalence of diabetes mellitus was 4% and 2% for women and men, while the prevalence of estimated glomerular filtration rate (eGFR) ≤60 ml/min/1.73m^2^ was 18% and 12% for women and men respectively (Table 2).

**Table 2:** Patient characteristics at the start of MMD (*N* = 1,082).

Characteristics	Women (*n* = 677)	Men (*n* = 405)	*P* value
Mean Age in yrs., (SD)	49.7 (9.4)	53.0 (9.4)	0.002
Age categories, yrs. (%)			
18–29	2 (0.3)	1 (0.2)	0.001
30–39	84 (12.4)	25 (6.2)	
40–49	250 (36.9)	124 (30.6)	
≥50	341 (50.4)	255 (63.0)	
BP in mmHg, (SD)			
Mean systolic BP	133.1 (15.8)	137.7 (14.8)	<0.0001
Mean diastolic BP	86.5 (9.9)	86.7 (10.8)	0.074
% HTN control (Absolute number)	77.1% (521)	67.6% (273)	0.001
BMI in kg/m^2^, (%)			
Underweight (<19.0)	30 (4.4)	27 (6.7)	0.213
Normal weight (19.0 to <25.0)	217 (32.1)	213 (52.6)	
Overweight (25.0 to <30.0)	237 (35.0)	125 (30.8)	
Obese (>30.0)	193 (28.5)	40 (9.9)	
ART regimen (%)			
TDF-3TC-DTG	486 (71.8)	323 (79.7)	0.005
TDF-3TC-EFV	85 (12.6)	24 (5.9)	
ABC-3TC-EFV	31 (4.6)	13 (3.2)	
OTHERS	75 (11.0)	45 (11.2)	
Duration on ART (%)			
<2 years	10 (1.5)	14 (3.5)	0.114
3 to <5years	76 (11.2)	42 (10.3)	
5 to <10years	256 (37.8)	176 (43.5)	
≥10years	335 (49.5)	173 (42.7)	
Other comorbidities (%)			
Diabetes mellitus	39 (3.6)	25 (2.3)	0.593
CKD - eGFR ≤60 ml/min/1.73m^2^	*n* = 257	*n* = 149	
CKD Stage 4/5 (0–30)	2 (0.8)	2 (1.3)	0.213
CKD Stage 3 (30–60)	44 (17.1)	16 (10.7)	

*BMI* Body mass index, *ART* Antiretroviral therapy, *eGRF* Estimated Glomerular filtration rate, *HTN* Hypertension, *CKD* Chronic Kidney Disease, *TDF* Tenofovir, *3TC* Lamivudine, *DTG* Dolutegravir, *ABC* Abacavir, *EFV* Efavirenz.

At the initial MMD, the majority (98.9%) of 1,082 had controlled HIV, while hypertension control was at 73.5%. The average age was 51 (SD = 9) years and 677 (63%) were females (Table 3).

**Table 3:** Hypertension and HIV treatment outcomes (*N* = 1,043).

Outcome	Baseline (Start of MMD)	12 months of MMD	*P* value
Mean systolic blood pressure, mmHg (SD)	135 (15.6)	132 (15.2)	<0.0001
Mean diastolic blood pressure, mmHg (SD)	87 (15.2)	86 (10.5)	0.021
Patients with controlled hypertension (%)	767 (73.5%)	817 (78.3%)	0.01
Patients with controlled HIV (Viral load <1000 copies/ml) (%)	1032 (98.9%)	1036 (99%)	0.65

There was a statistically significant reduction in both the mean systolic blood pressure (135 vs 132 mmHg, *p* < 0.0001) and the mean diastolic blood pressure (87 vs 86 mmHg, *p* = 0.021) from baseline and at 12 months. The proportion of patients with controlled blood pressure improved from 767 (73.5%) at the start of MMD to 817 (78.3%) at 12 months (*p* = 0.01). Of the 767 patients that had controlled blood pressure at baseline, 637 (83%) sustained blood pressure control at 12 months. In addition, there was no significant change in the proportion of patients with controlled HIV at baseline 1,032 (98.9%) and after 12 months 1036 (99%) (*p* = 0.65). Providing integrated MMD for hypertension and ART improved hypertension control without disrupting HIV viral suppression (Table 3).

**Fig. 2:**
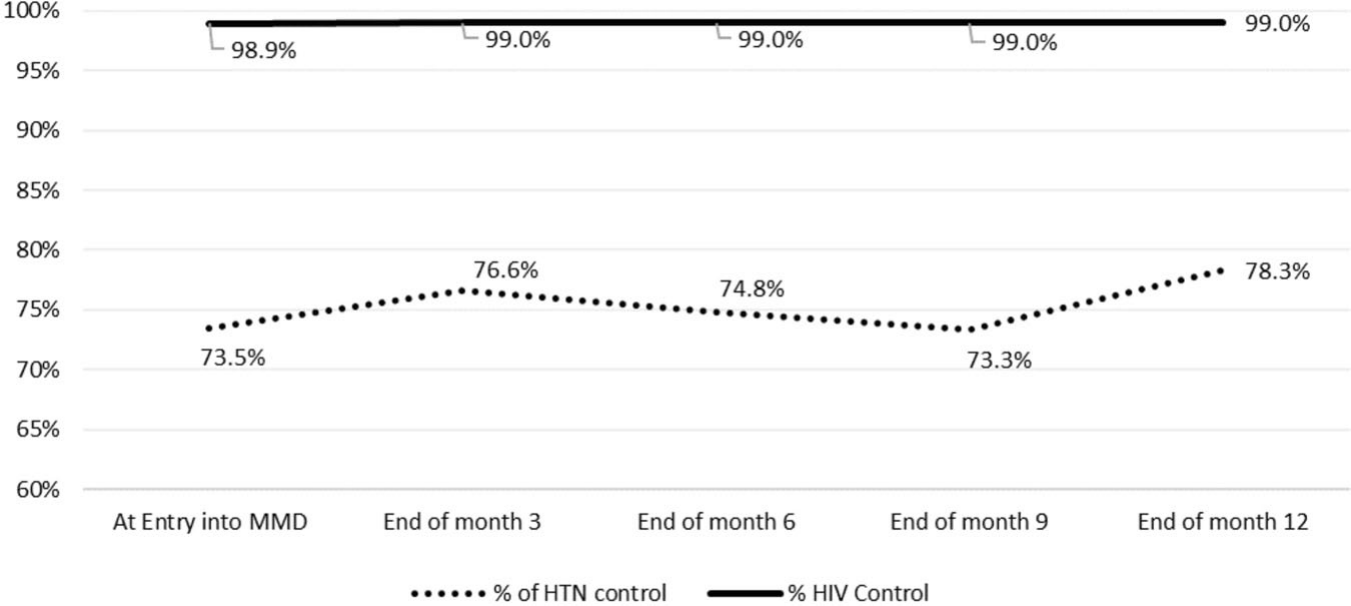
Longitudinal trend of hypertension and HIV control during the 12 month follow-up. The proportions are percentages of hypertensive PLHIV on multi-month dispensing with controlled hypertension and HIV.

Hypertensive PLHIV on MMD sustained HIV control (HIV viral load of >98%) through the 12 months of follow-up. There was a general trend of improvement in hypertension control from the start of MMD through the four quarters of the 12 months. The decrease in hypertension control midway on MMD corresponded to the period of Covid-19 related national lockdown and travel restrictions (Fig. 2).

After 12 months of follow-up, 1,043 (96.4%) patients were retained on integrated MMD and four were lost to follow-up. Majority, 751 (98%) of the patients that had controlled blood pressure at baseline were retained in care. Twelve (1.1%) patients requested to be transferred to health facilities closer to their homes, mainly due to increased transport costs during the Covid-19 pandemic. Eight (0.7%) patients died with a mean age of 56 years: of these, five were male, and three female. The causes of death were hepatic carcinoma (*n* = 2), stroke (*n* = 2), pulmonary tuberculosis (*n* = 1), throat cancer (*n* = 1), renal failure (*n* = 1) and heart attack (*n* = 1). All patients died from the hospital, and the causes of death were recorded from the death certificates that were presented by the relatives. The patients who died of a stroke succumbed during the period of national lockdown and movement restriction to prevent the spread of COVID-19: they had no access to hypertension medications.

We had 15 (1.4%) patients opting out of integrated MMD due to hypertension drug adverse events that included lower limb edema (*n* = 4), palpitations (*n* = 4), persistent dizziness (*n* = 3), hypersensitivity skin reactions (*n* = 2) and persistent headaches (*n* = 2).

Patients on ART for more than five years were more likely to have controlled hypertension at 12 months. However, females, patients with elevated systolic blood pressure at baseline, and those on ABC-3TC-EFV ART regimen were less likely to have controlled blood pressure at 12 months (Table 4).

**Table 4:** Predictors of blood pressure control while receiving MMD (*N* = 1,043).

Category	Adjusted Odds Ratio	95% CI	*P* value
Gender			
Females	0.696	0.534–0.906	0.007
Age, years			
18–29 (Reference)			
30–39	1.586	0.285–8.823	0.598
40–49	1.847	0.342–9.974	0.476
≥50	1.831	0.341–9.835	0.481
BP ranges			
Systolic blood pressure	0.914	0.903–0.925	<0.0001
Diastolic blood pressure	0.994	0.977–1.010	0.473
Duration on ART			
<2 years (Reference)			
3 to <5years	2.35	0.815–9.775	0.113
5 to <10years	2.78	1.051–7.324	0.039
≥10years	3.36	1.277–8.821	0.014
Diabetes status			
Non-diabetic	0.85	0.476–1.530	0.594
ART regimen			
TDF-3TC-DTG (Reference)			
TDF-3TC-EFV	0.712	0.463–1.097	0.124
ABC-3TC-EFV	0.293	0.150–0.572	<0.0001
OTHERS	0.894	0.591–1.352	0.597

## Discussion

Implementing an integrated MMD strategy for both antihypertensive medicines and ART significantly improved blood pressure control from 73.5% at baseline to 78.3% at 12 months of follow-up. Importantly, this improvement in hypertension control happened without disrupting HIV control (>98% HIV viral suppression throughout the 12 months of follow-up). Therefore, integrated MMD of both hypertension medications and ART among hypertensive PLHIV improved hypertension and maintained HIV control. Walsh et al., also reported improved blood pressure control in integrated hypertension-HIV care [11].

In this study, there was a 17% drop in hypertension control at 12 months in patients with controlled blood pressure at baseline. This drop rate was lower than the 21% at 12 months reported by Birtwhistle and colleagues, among patients randomized to receive three monthly hypertension drug refills [24].

Across the four quarters of follow-up, we observed a general trend of improvement in hypertension control and sustained HIV control. However, there was a decline in hypertension control amidst sustained excellent HIV control midway of follow-up, which happened during the period of national lockdown and movement restriction in Uganda to prevent the spread of Covid-19 (Fig. 2). At that time, patients who had been receiving integrated hypertension-HIV care at our clinic, who were unable to come to the clinic due to the lockdown, could easily access ART but not hypertension medicines at alternative HIV clinics. Hypertension medications were not available in many of the HIV clinics that our patients visited. The national lockdown unmasked a health system weakness of a vertical (disease-centered) approach to chronic disease management. Other than Mulago ISS, many HIV clinics in the country had not yet integrated the services of HIV and hypertension care [25].

Overall, 96% of patients were retained in care at one year while receiving integrated MMD for both hypertension and HIV medications. The overall retention in care was lower than that of patients with both controlled HIV and hypertension at baseline, which was at 98%. However, the retention in our cohort was higher than that in the general HIV population of about 76% [26] and 86% for stable patients [27]. Integrated MMD of both hypertension and ART medications in our cohort reduced the frequency of clinic visits, transportation costs and minimized patient loss to follow-up and improved retention in care as well as HIV and hypertension control. In other studies across SSA, MMD facilitates retention of patients in care, because it reduces the patients’ travel and time cost [17, 18].

Twelve (1.1%) patients who requested to transfer to a different health facility cited high transport costs as the major reason, especially during the Covid-19 movement restrictions. Geng et al. reported that structural barriers like transportation challenges contributed most to patients who get lost to follow-up and silent transfers [21]. Fifteen (1.4%) patients opted out of integrated hypertension-HIV care due to hypertension medicine-related adverse effects of lower limb swelling and palpitations. These patients opted for alternative medications for hypertension but remained active in care at the HIV clinic. This prevalence (1.4%) of adverse drug events necessitating a drug change was lower than the frequency reported in literature of about 3-4% [20]. Two of the four patients that died from a cardiovascular disease related cause died during the period of interrupted hypertension treatment due to the Covid-19 national lockdown, and four of the eight deaths were not cardiovascular related. Based on the optimal retention, good HIV and hypertension control as well as minimal side effects of the treatment in our cohort, the integrated MMD approach and the hypertension protocol were safe for patients.

At baseline, most of the patients (80%) had been on ART for more than five years, and were more likely to have controlled hypertension at 12 months compared to those with a shorter duration on ART. Patients with longer than five years on ART may have adjusted better to lifelong treatment through adherence counseling and support for ART, and could easily adhere to additional hypertension medicines. In the SEARCH trial, patients with hypertension and HIV co-morbidity who received integrated care were more likely to have controlled hypertension than those with hypertension alone. The major contributor to hypertension control among hypertensive PLHIV in this study was extensive drug adherence counseling [28].

Female patients and those with elevated systolic blood pressure at baseline were less likely to have controlled blood pressures at 12 months. Since high blood pressure at baseline is a negative predictor of hypertension control among patients who received MMD, it is implied that patients with controlled blood pressure would benefit more from integrated MMD than those with uncontrolled blood pressure. Therefore, integrated hypertension-HIV programs should prioritize patients with both controlled hypertension and HIV for MMD.

Compared to 9.9% of the men, 28.5% of women were obese (BMI > 30 kg/m²). Obesity is a strong risk factor for resistant hypertension [29], which may explain the lower likelihood of controlled hypertension in females at 12 months. The high prevalence of obesity, a modifiable cardiovascular disease risk factor, among females in this cohort calls for routine healthy lifestyle counseling. Patients on ABC-3TC-EFV regimen were also less likely to have controlled hypertension. Abacavir-based ART regimens are commonly given as alternatives to patients with chronic kidney disease (CKD) in whom the preferred first line Tenofovir is contraindicated. CKD is a common cause and complication of poor hypertension control and may explain lower hypertension control in patients on Abacavir [29]. Overall, the prevalence of CKD in our cohort was 16%.

Despite successful implementation of MMD in the HIV program, it has not been fully embraced for other chronic diseases like hypertension. MMD depends on a robust medicine supply chain if stockouts are to be averted. The HIV supply chain in Uganda and other SSA settings is robust and reliable due to the great efforts of national Ministries of Health and PEPFAR [26]. However, the medicine supply chains for chronic non-communicable diseases (NCDs) across SSA are still weak [30]. To prevent frequent stock out of medicines for NCDs given the weak supply chains, health systems opt for monthly patient visits and dispensing for hypertension and other NCDs. Monthly drug dispensing comes at a cost of increased transport needs to patients, poor adherence to medications, high level of loss to follow-up, and poor hypertension control [31].

In this study, one clinic pharmacist utilized the ministry of health recommended electronic logistics information systems for medicines management to forecast, quantify and monitor stock levels for both ART and hypertension medicines [32]. This facilitated timely placement of orders to replenish stocks of both ART and antihypertensives medicines. Integrated management of supply chains for ART and hypertension medications was feasible but utilized existing HIV capacity and infrastructure to avoid both ART and hypertension medicines stockouts. This underscores a need for innovative integration of chronic management of HIV and NCDs by leveraging and consolidating the gains of the HIV program.

As HIV programs continue to scale up extended dispensing intervals of up to six months for stable patients, remaining evidence gaps in integrated MMD would require controlled longitudinal studies to determine the most optimal dispensing intervals, their cost, and cost-effectiveness.

We acknowledge that there are limitations to this study. First, we conducted the study in a successful urban HIV clinic meeting all the 90-90-90 UNAIDS targets, which may not be representative of some HIV clinics that are performing sub-optimally on the above targets. Second, participants were consecutively enrolled into MMD as and when they became stable on both hypertension and HIV treatment, without a control group. However, our pre-post prospective study controlled for time-varying confounders by comparing the study outcomes at baseline and 12 months of follow-up. Thus, our integrated MMD findings would be generalizable to stable hypertensive PLHIV.

In summary, we demonstrated the feasibility of an integrated MMD strategy for HIV and hypertension therapy, with the potential to improve hypertension control that could be modeled for the treatment of other chronic conditions in SSA and elsewhere.

### Summary

#### What is known about the topic

- High prevalence of cardiovascular disease among persons living with HIV
- Adequate hypertension control reduces cardiovascular disease mortality and morbidity

#### What this study adds

- Feasibility of integrated multi-month dispensing (MMD) for both HIV and hypertension medicines among hypertensive persons living with HIV
- Integrated MMD for both HIV and hypertension medications improved hypertension control and sustained excellent HIV control among hypertensive persons living with HIV

## Data Availability

The datasets used and/or analysed that support the findings of this study are available from the corresponding author on reasonable request.
